# Cardiometabolic biomarker profiles associated with estimated 10-year ASCVD risk by the China-PAR model in an older adult health examination cohort: a cross-sectional study

**DOI:** 10.3389/fmed.2026.1897876

**Published:** 2026-10-14

**Authors:** LiYing Zhang, Pu Chen, WenTao Zhou, WeiLei Xiao

**Affiliations:** 1 Department of Clinical Laboratory, The First People’s Hospital of Yibin, Yibin, Sichuan, China; 2 Department of Clinical Laboratory, The Third People’s Hospital of Yibin City, Yibin, Sichuan, China; 3 School of Laboratory Medicine, North Sichuan Medical College, Nanchong, Sichuan, China

**Keywords:** atherogenic coefficient, atherosclerotic cardiovascular disease, biomarkers, China-PAR, cystatin C, fasting plasma glucose, NT-proBNP, older adults

## Abstract

**Objective:**

To characterize the associations between cardiometabolic biomarker profiles and estimated 10-year atherosclerotic cardiovascular disease (ASCVD) risk calculated using the China-PAR (Prediction for Atherosclerotic Cardiovascular Disease Risk in China) equation in adults aged ≥60 years undergoing routine physical examinations.

**Methods:**

Participants aged ≥60 years were recruited from a hospital health examination center. Ten-year ASCVD risk was calculated using the China-PAR equation and classified as low-to-intermediate (<10%) or high (≥10%). Associations between continuous biomarkers and the risk score were assessed using Spearman correlation with false discovery rate correction. Exploratory LASSO logistic regression was used for feature selection for China-PAR-defined high-risk status, followed by mutually adjusted logistic regression and quartile-stratified analyses.

**Results:**

A total of 511 participants were included (median age 67 years; 62.8% male), and 191 (37.4%) were classified as high risk by China-PAR. Waist-to-hip ratio (WHR; rho = 0.43), fasting plasma glucose (FPG; rho = 0.40), and cystatin C (CysC; rho = 0.39) showed the strongest correlations with the calculated risk score. Exploratory LASSO feature selection retained CysC, FPG, atherogenic coefficient (AC), and N-terminal pro-B-type natriuretic peptide (NT-proBNP). Per one-standard-deviation increase, FPG [adjusted odds ratio [aOR] = 2.772, 95% confidence interval [CI]: 2.047–3.753] and CysC (aOR = 2.731, 95% CI: 1.881–3.964) showed the strongest associations with China-PAR-defined high-risk status. All four biomarkers showed graded trends across quartiles in the overall population and in descriptive sex-stratified analyses.

**Conclusions:**

CysC, FPG, AC, and NT-proBNP were associated with higher estimated China-PAR risk. Because FPG and AC overlap clinically or mathematically with variables used to construct China-PAR, their associations should be interpreted partly in the context of score construction. CysC and NT-proBNP are not components of the China-PAR equation and warrant prospective evaluation. These findings characterize biomarker profiles associated with higher estimated cardiovascular risk and may help increase clinical awareness of unfavorable cardiometabolic and cardiorenal features during routine health examinations.

## Introduction

With the rising prevalence of metabolic diseases such as obesity and diabetes, atherosclerotic cardiovascular disease (ASCVD) has become a major global public health concern (1). Cardiovascular disease remains the leading cause of death worldwide, with its incidence showing a persistent upward trend in recent years (2). Against this backdrop of an increasingly severe disease burden, accurate risk assessment is crucial for the effective prevention and management of ASCVD. Importantly, older adults (aged ≥ 60 years) represent a core high-risk segment for ASCVD, in whom early identification and risk stratification are particularly relevant for primary prevention. Consequently, the development and refinement of reliable risk prediction tools have become a significant focus in cardiovascular research.

In China, the earliest risk prediction equations for coronary heart disease and ischemic cardiovascular disease were established 14 years ago (3). However, China has undergone rapid economic development and significant demographic shifts over recent decades, necessitating an update to its cardiovascular risk assessment framework. Initially, the lack of a dedicated risk assessment tool for the Chinese population led to the widespread use of foreign risk assessment tools in large-scale Chinese surveys, which may lead to an over- or underestimation of overall cardiovascular risk (4). To address this issue and provide timely and accurate information for the prevention and management of cardiovascular disease in China, the Prediction for ASCVD Risk in China (China-PAR) equation was developed (5). This 10-year ASCVD risk prediction model is now recommended by the *Guideline on the Assessment and Management of Cardiovascular Risk in China*, representing a significant advancement in risk assessment for the Chinese population (5). The China-PAR equation, derived from recent cohort data and validated in two independent Chinese cohorts, demonstrates superior predictive performance compared to earlier models. Furthermore, given that different types of ASCVD share similar risk factors and pathophysiological mechanisms, the China-PAR framework provides a solid foundation for comprehensive cardiovascular risk assessment in the Chinese population (6).

The China-PAR model has demonstrated acceptable performance in its original validation and subsequent studies (7). Beyond the variables incorporated into the equation, routinely measured laboratory biomarkers may characterize cardiometabolic phenotypes that accompany higher estimated risk (8, 9). However, associations with a formula-derived risk score should be distinguished from incremental prognostic value for clinical events (10, 11). Evaluating biomarker patterns alongside the calculated score may nevertheless help describe the clinical phenotype of older adults classified into different China-PAR risk strata (12).

Older adults undergoing routine health checkups are a key target for primary cardiovascular prevention, typically at an early or asymptomatic stage, making it crucial for early risk identification and stratified management (13). Compared to clinical patients, this population shows a continuous spectrum of risk levels ranging from low to high, thus relying more heavily on quantitative risk assessment to guide individualized prevention strategies. As a validated ASCVD risk prediction tool developed for the Chinese population, China-PAR provides a 10-year risk assessment equation, offering a methodological basis for primary prevention and management in the general population (5). However, existing research on the application of China-PAR in health checkup settings has largely focused on describing risk distribution and stratification features, or has only analyzed the association between a limited number of laboratory indices/ratios [e.g., total cholesterol [TC]/high-density lipoprotein cholesterol [HDL-C]] and risk categories (14). Current studies often concentrate on the association between a few laboratory indicators (e.g., uric acid/hyperuricemia) and China-PAR risk levels, highlighting a relative lack of research that comprehensively incorporates multiple indicators for a profile-based assessment.

Therefore, the primary aim of this cross-sectional study was to characterize the associations between a broad cardiometabolic biomarker profile and estimated 10-year ASCVD risk calculated by China-PAR in adults aged ≥60 years undergoing routine physical examinations. A secondary exploratory aim was to identify a parsimonious set of biomarkers associated with China-PAR-defined high-risk status and to describe their quartile-based distributions. The study was not designed to develop or validate a prognostic model for incident ASCVD events, but rather to identify biomarker features that may provide clinically relevant context for interpreting higher estimated cardiovascular risk during routine health examinations.

## Materials and methods

### Study design and participants

This was a cross-sectional study that enrolled individuals undergoing routine health examinations at our hospital's health checkup center from May to August 2025. The inclusion criteria were: (1) age ≥60 years; (2) completion of a comprehensive physical examination and laboratory tests; (3) availability of complete data required for China-PAR risk calculation. The exclusion criteria were: (1) a history of diagnosed ASCVD, including myocardial infarction, coronary revascularization, stroke, or peripheral arterial disease; (2) severe hepatic or renal dysfunction; (3) malignant tumors or other life-threatening diseases; (4) incomplete clinical or laboratory data. Among 566 individuals initially assessed, 55 were excluded because of incomplete data, leaving 511 participants in the final analytic cohort (Figure 1). Missingness predominantly involved hematologic and platelet-related measurements: 49 records had missing hematologic/platelet measurements, five had missing platelet indices only, and one had missing WHR. Records with incomplete data were excluded by design; no imputation was performed. The study protocol was approved by the Institutional Ethics Committee of The First People's Hospital of Yibin, and the requirement for written informed consent was waived by the Ethics Committee.

**Figure 1:**
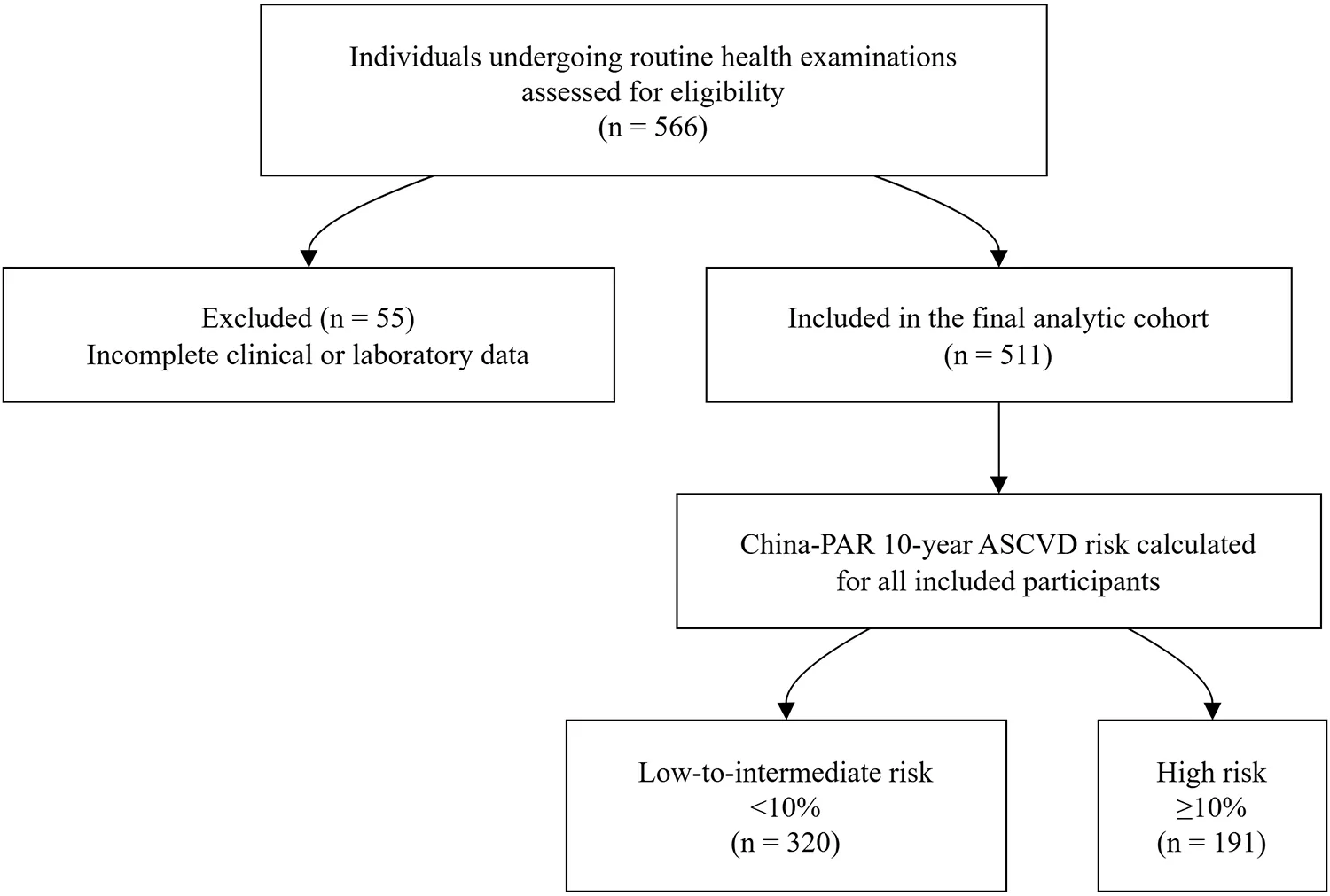
Participant flow diagram. Among 566 individuals initially assessed, 55 were excluded because of incomplete clinical or laboratory data. Missingness involved hematologic/platelet measurements in 49 records, platelet indices only in five records, and WHR in one record. The final analytic cohort comprised 511 participants with complete data for the reported analyses. WHR, waist-to-hip ratio.

### Data collection

Demographic characteristics, clinical data, and laboratory results were systematically collected for all participants. Demographic and clinical characteristics included age, sex, region (Northern or Southern China), type of residence (urban or rural), current smoking status, diabetes, use of antihypertensive medication, personal medical history, and family history of cardiovascular disease. Current smoking was defined as smoking at least one cigarette per day. Diabetes was defined as a fasting plasma glucose (FPG) level ≥7.0 mmol/L, a prior physician diagnosis, or current use of glucose-lowering medication. A family history of cardiovascular disease was defined as coronary heart disease or stroke in a first-degree relative.

### Physical examination

Trained medical staff conducted physical examinations following standardized procedures. Anthropometric measurements included waist and hip circumferences, recorded to the nearest 0.1 cm. The waist-to-hip ratio (WHR) was calculated. Blood pressure was measured using an automatic sphygmomanometer after the participant had rested in a seated position for at least 5 min. Systolic blood pressure (SBP) and diastolic blood pressure (DBP) were recorded as the average of two consecutive measurements.

### Laboratory measurements

Venous blood samples were collected after a minimum 8 h fast and analyzed using standardized methods. Cardiovascular markers included lipoprotein(a) [Lp(a)], small dense low-density lipoprotein cholesterol (sdLDL-C), homocysteine (HCY), high-sensitivity cardiac troponin I (hs-cTnI), and N-terminal pro-B-type natriuretic peptide (NT-proBNP). Lipid profiles comprised TC, HDL-C, low-density lipoprotein cholesterol (LDL-C), and triglycerides (TG). The atherogenic coefficient (AC) was calculated as AC = (TC − HDL-C) / HDL-C, equivalent to the non-HDL-C/HDL-C ratio. The glycemic metabolism indicator was fasting plasma glucose (FPG). Renal function markers included serum urea, creatinine, uric acid (UA), and cystatin C (CysC). Complete blood count parameters included white blood cell count (WBC), percentages of neutrophils (NEUT%), eosinophils (EOS%), basophils (BASO%), lymphocytes (LYM%), and monocytes (MONO%), along with their absolute counts (NEUT_abs, EOS_abs, BASO_abs, LYM_abs, MONO_abs); red blood cell count (RBC), hemoglobin (HGB), hematocrit (HCT), mean corpuscular volume (MCV), mean corpuscular hemoglobin (MCH), mean corpuscular hemoglobin concentration (MCHC), red cell distribution width (RDW-SD and RDW-CV); platelet count (PLT), plateletcrit (PCT), mean platelet volume (MPV), platelet distribution width (PDW), and large platelet ratio (P-LCR%). Liver biochemical indicators included, total bilirubin (TBIL), indirect bilirubin (IBIL), alanine aminotransferase (ALT), aspartate aminotransferase (AST), gamma-glutamyl transferase (GGT), alkaline phosphatase (ALP), total protein (TP), albumin (ALB), globulin (GLO), and the albumin-to-globulin ratio (A/G ratio). All laboratory tests were conducted following standardized procedures and the quality control was fully within the acceptable range.

### China-PAR risk assessment

The 10-year ASCVD risk was calculated for each participant using the China-PAR equation (available at: https://www.cvdrisk.com.cn/ASCVD/Eval). The China-PAR model estimates the 10-year risk of developing ASCVD, defined as non-fatal myocardial infarction, death from coronary heart disease, or fatal or non-fatal stroke. This model incorporates variables including age, sex, region, type of residence, waist circumference, SBP, TC, HDL-C, current smoking status, diabetes, use of antihypertensive medication, and family history of cardiovascular disease. Risk scores are expressed as percentages and categorized into three levels based on established thresholds: low risk (<5%), intermediate risk (5% to <10%), and high risk (≥10%). For the main group comparisons and logistic analyses, low and intermediate categories were combined (<10%) to contrast participants below vs. at or above the 10% high-risk threshold. The continuous China-PAR percentage was retained for correlation and quartile analyses.

### Statistical analysis

Continuous variables are presented as median (interquartile range, IQR), and categorical variables are presented as frequencies and percentages. Baseline comparisons between the low-to-intermediate and high-risk groups were performed using the Mann–Whitney *U* test for continuous variables and the chi-square test or Fisher's exact test for categorical variables, as appropriate. Associations between continuous biomarkers and the continuous China-PAR risk score were evaluated using Spearman rank correlation. Because multiple biomarker correlations were examined, false discovery rate (FDR)-adjusted *q* values were reported in addition to two-sided *P* values.

Exploratory least absolute shrinkage and selection operator (LASSO) logistic regression with a binomial outcome was used to select laboratory features associated with China-PAR-defined high-risk status (≥10%). A total of 47 eligible continuous laboratory variables were entered after excluding identifiers and variables used directly in the China-PAR equation. Predictors were standardized internally during model fitting by glmnet. The penalty parameter (*λ*) was selected by 10-fold cross-validation using area under the receiver operating characteristic curve (AUC), with lambda.min used for the primary feature-selection solution and lambda.1se inspected as a more parsimonious reference. Variable-selection stability was additionally assessed using 1,000 bootstrap resamples; in each resample, the complete cross-validated LASSO procedure was refitted and selection at the resample-specific lambda.min was recorded. The LASSO analysis was treated as exploratory feature selection for a formula-derived risk category and not as evidence of incremental prediction of future ASCVD events.

The features retained at lambda.min were subsequently entered into univariate and mutually adjusted logistic regression models. For these logistic regression analyses, NT-proBNP was natural-log transformed before *z*-score standardization because of its skewed distribution; the other selected biomarkers were *z*-score standardized on their original scales. Odds ratios (ORs) represent the change in odds of China-PAR-defined high-risk status per one-standard-deviation increase in each biomarker. The multivariable model adjusted the selected biomarkers for one another only; China-PAR components were not added as covariates because those variables mathematically define the outcome. For quartile analyses, participants were divided into Q1–Q4 according to each selected biomarker. Linear trend tests assessed changes in the continuous risk score across quartiles, and tests for trend in proportions assessed the high-risk proportion. Sex-stratified analyses were descriptive; within-sex *P* values were not interpreted as formal tests of biomarker-by-sex interaction.

All statistical analyses were performed using R software (version 4.3.0). LASSO regression was conducted using the glmnet package. A two-sided *P*-value <0.05 was considered statistically significant; FDR-adjusted *q* values were used for the multiple correlation analysis.

## Results

### Baseline characteristics of the study population

Among 566 individuals initially assessed, 55 were excluded because of incomplete clinical or laboratory data, leaving 511 individuals aged ≥60 years in the final analytic cohort (Figure 1). The final cohort comprised 321 males (62.8%) and had a median age of 67 years (interquartile range: 62–71). Based on their China-PAR scores, participants were categorized into a low-to-intermediate risk group (<10%, *n* = 320) and a high-risk group (≥10%, *n* = 191). Table 1 presents a comparison of the baseline characteristics between these two groups. The high-risk group had a significantly higher proportion of males (77.0% vs. 54.4%, *P* < 0.001) and was older (72 vs. 63 years, *P* < 0.001); it also exhibited significantly higher rates of antihypertensive medication use (24.1% vs. 13.8%, *P* = 0.003) and diabetes prevalence (49.7% vs. 10.6%, *P* < 0.001). Regarding anthropometric measures, the high-risk group showed higher values for waist circumference (88 vs. 83 cm, *P* < 0.001), SBP (144 vs. 126 mmHg, *P* < 0.001), DBP (78 vs. 74 mmHg, *P* = 0.004), and WHR (0.93 vs. 0.89, *P* < 0.001). For lipid profiles, the high-risk group exhibited lower HDL-C (1.09 vs. 1.31 mmol/L, *P* < 0.001) and higher TG (1.66 vs. 1.29 mmol/L, *P* < 0.001). Among novel cardiovascular markers, the high-risk group demonstrated elevated levels of sdLDL-C (0.48 vs. 0.42 mmol/L, *P* = 0.027), HCY (13.63 vs. 12.04 μmol/L, *P* = 0.005), hs-cTnI (2.08 vs. 1.44 ng/L, *P* < 0.001), and NT-proBNP (58.26 vs. 41.09 pg/mL, *P* < 0.001). Renal function markers, including creatinine, UA, and CysC (1.16 vs. 1.03 mg/L, *P* < 0.001), were higher in the high-risk group. The AC (3.37 vs. 2.59, *P* < 0.001) and FPG (6.33 vs. 5.39 mmol/L, *P* < 0.001) were also higher in the high-risk group. In the complete blood count, the high-risk group had a higher neutrophil percentage (59.30% vs. 56.35%, *P* < 0.001), a lower LYM percentage (30.90% vs. 34.30%, *P* < 0.001), and a reduced PLT (180 × 10^9^/L vs. 195 × 10^9^/L, *P* = 0.017). Among liver function indicators, GGT was significantly elevated in the high-risk group (28 vs. 23 U/L, *P* < 0.001).

**Table 1:** Comparison of baseline characteristics between the low/medium-risk and high-risk groups.

Variable	Level	Overall (*N* = 511)	Low/Medium (*N* = 320)	High (*N* = 191)	*P* value
Sample size					
*N*		511	320	191	
Demographics					
Age, years		67.00 (62.00, 71.00)	63.00 (61.00, 68.00)	72.00 (67.00, 77.00)	<0.001
Sex	Female	190 (37.2%)	146 (45.6%)	44 (23.0%)	<0.001
	Male	321 (62.8%)	174 (54.4%)	147 (77.0%)	
Region	North	7 (1.4%)	5 (1.6%)	2 (1.0%)	1.000
	South	504 (98.6%)	315 (98.4%)	189 (99.0%)	
Residence	Rural	83 (16.2%)	57 (17.8%)	26 (13.6%)	0.213
	Urban	428 (83.8%)	263 (82.2%)	165 (86.4%)	
Medical history & lifestyle					
Current smoking	No	379 (74.2%)	242 (75.6%)	137 (71.7%)	0.330
	Yes	132 (25.8%)	78 (24.4%)	54 (28.3%)	
Antihypertensive medication use	No	421 (82.4%)	276 (86.2%)	145 (75.9%)	0.003
	Yes	90 (17.6%)	44 (13.8%)	46 (24.1%)	
Diabetes	No	382 (74.8%)	286 (89.4%)	96 (50.3%)	<0.001
	Yes	129 (25.2%)	34 (10.6%)	95 (49.7%)	
History of disease (any)	No	260 (50.9%)	194 (60.6%)	66 (34.6%)	<0.001
	Yes	251 (49.1%)	126 (39.4%)	125 (65.4%)	
Family history (any)	No	448 (87.7%)	274 (85.6%)	174 (91.1%)	0.069
	Yes	63 (12.3%)	46 (14.4%)	17 (8.9%)	
Anthropometrics					
Waist circumference		85.00 (80.00, 91.00)	83.00 (77.00, 88.25)	88.00 (83.00, 93.00)	<0.001
Hip circumference		93.00 (89.00, 97.00)	93.00 (89.00, 97.00)	94.00 (90.00, 98.00)	0.263
Waist-to-hip ratio (WHR)		0.91 (0.86, 0.95)	0.89 (0.85, 0.93)	0.93 (0.90, 0.97)	<0.001
Blood pressure					
Systolic blood pressure (SBP)		134.00 (122.00, 146.00)	126.00 (116.00, 136.50)	144.00 (136.00, 154.00)	<0.001
Diastolic blood pressure (DBP)		76.00 (70.00, 82.00)	74.00 (68.00, 82.00)	78.00 (70.00, 85.00)	0.004
Lipids & atherogenic markers					
Total cholesterol (TC)		4.69 (4.13, 5.47)	4.72 (4.13, 5.48)	4.65 (4.13, 5.46)	0.738
Triglycerides (TG)		1.44 (1.02, 2.02)	1.29 (0.96, 1.74)	1.66 (1.17, 2.25)	<0.001
HDL cholesterol (HDL-C)		1.24 (0.99, 1.50)	1.31 (1.09, 1.62)	1.09 (0.89, 1.32)	<0.001
LDL cholesterol (LDL-C)		2.85 (2.25, 3.42)	2.83 (2.26, 3.38)	2.90 (2.25, 3.49)	0.366
Small dense LDL cholesterol (sdLDL-C)		0.43 (0.27, 0.62)	0.42 (0.27, 0.58)	0.48 (0.27, 0.69)	0.027
Lipoprotein(a) [Lp(a)]		9.79 (5.15, 28.49)	9.80 (5.28, 27.63)	9.39 (4.90, 30.12)	0.384
Atherogenic coefficient (AC)		2.75 (1.83, 3.79)	2.59 (1.65, 3.48)	3.37 (2.19, 4.17)	<0.001
Glucose metabolism					
Fasting plasma glucose (FPG)		5.52 (5.03, 6.54)	5.39 (4.99, 5.83)	6.33 (5.24, 7.81)	<0.001
Renal function					
Serum creatinine (Scr)		71.30 (59.05, 83.10)	68.30 (57.45, 80.53)	74.60 (64.75, 86.65)	<0.001
Cystatin C (CysC)		1.07 (0.96, 1.20)	1.03 (0.93, 1.13)	1.16 (1.04, 1.31)	<0.001
Urea		5.61 (4.71, 6.54)	5.58 (4.72, 6.51)	5.63 (4.70, 6.62)	0.588
Uric acid (UA)		342.00 (288.00, 402.00)	333.50 (282.75, 392.00)	360.00 (300.00, 428.00)	<0.001
Cardiac biomarkers					
High-sensitivity cardiac troponin I (hs-cTnI)		1.71 (1.12, 2.63)	1.44 (1.05, 2.27)	2.08 (1.46, 3.16)	<0.001
N-terminal pro-B-type natriuretic peptide (NT-proBNP)		45.46 (26.98, 72.33)	41.09 (23.32, 61.55)	58.26 (31.84, 98.33)	<0.001
Hematology					
White blood cell count (WBC)		6.04 (5.19, 7.13)	5.96 (5.06, 7.00)	6.22 (5.41, 7.33)	0.031
Red blood cell count (RBC)		4.71 (4.33, 5.06)	4.69 (4.32, 5.01)	4.77 (4.42, 5.12)	0.084
Hemoglobin (HGB)		141.00 (132.00, 151.00)	140.00 (131.00, 150.00)	144.00 (132.00, 152.50)	0.111
Hematocrit (HCT)		43.70 (40.70, 46.50)	43.40 (40.68, 46.40)	44.20 (40.80, 46.60)	0.271
Mean corpuscular volume (MCV)		93.20 (90.35, 96.00)	93.55 (90.60, 96.20)	92.70 (90.00, 95.50)	0.146
Mean corpuscular hemoglobin (MCH)		30.30 (29.30, 31.20)	30.30 (29.30, 31.20)	30.20 (29.20, 31.20)	0.668
Mean corpuscular hemoglobin concentration (MCHC)		323.00 (319.00, 328.00)	323.00 (318.00, 328.25)	324.00 (319.00, 328.00)	0.187
Red cell distribution width-SD (RDW-SD)		45.20 (43.10, 46.95)	45.15 (42.98, 47.00)	45.20 (43.25, 46.80)	0.954
Red cell distribution width-CV (RDW-CV)		13.20 (12.80, 13.60)	13.10 (12.80, 13.60)	13.20 (12.80, 13.60)	0.373
Platelet count (PLT)		191.00 (156.00, 228.00)	195.00 (159.75, 232.25)	180.00 (154.00, 220.00)	0.017
Plateletcrit (PCT)		0.21 (0.18, 0.24)	0.22 (0.18, 0.25)	0.20 (0.17, 0.23)	0.005
Mean platelet volume (MPV)		11.00 (10.05, 12.00)	11.00 (10.00, 12.00)	11.10 (10.25, 12.00)	0.537
Platelet distribution width (PDW)		16.30 (15.75, 16.60)	16.20 (15.70, 16.52)	16.50 (16.00, 16.70)	<0.001
Platelet large cell ratio (P-LCR)		32.90 (26.05, 40.40)	32.50 (25.08, 40.82)	33.70 (27.30, 40.05)	0.371
Leukocyte differential					
Neutrophils (%)		57.10 (51.60, 62.80)	56.35 (51.08, 61.65)	59.30 (52.50, 64.50)	<0.001
Lymphocytes (%)		33.20 (27.35, 38.40)	34.30 (28.58, 38.90)	30.90 (25.70, 37.05)	<0.001
Monocytes (%)		6.20 (5.40, 7.30)	6.20 (5.40, 7.30)	6.30 (5.50, 7.35)	0.349
Eosinophils (%)		2.20 (1.40, 3.40)	2.20 (1.40, 3.40)	2.10 (1.30, 3.50)	0.999
Basophils (%)		0.40 (0.30, 0.60)	0.50 (0.30, 0.60)	0.40 (0.30, 0.60)	0.449
Lymphocyte count		1.96 (1.58, 2.43)	1.98 (1.62, 2.42)	1.94 (1.50, 2.44)	0.300
Monocyte count		0.39 (0.30, 0.48)	0.38 (0.30, 0.46)	0.41 (0.31, 0.50)	0.021
Eosinophil count		0.13 (0.08, 0.21)	0.13 (0.08, 0.21)	0.13 (0.08, 0.22)	0.570
Basophil count		0.03 (0.02, 0.04)	0.03 (0.02, 0.04)	0.03 (0.02, 0.04)	0.930
Neutrophil count		3.47 (2.84, 4.10)	3.37 (2.78, 3.91)	3.70 (3.00, 4.47)	<0.001
Liver function & proteins					
Alanine aminotransferase (ALT)		17.00 (12.00, 23.00)	17.00 (12.00, 23.00)	17.00 (12.00, 23.00)	0.800
Aspartate aminotransferase (AST)		20.00 (17.00, 24.00)	20.00 (17.00, 24.00)	20.00 (17.00, 24.00)	0.433
Gamma-glutamyl transferase (GGT)		25.00 (19.00, 36.00)	23.00 (18.00, 32.25)	28.00 (21.00, 40.00)	<0.001
Alkaline phosphatase (ALP)		75.00 (62.50, 92.00)	74.00 (61.75, 92.00)	77.00 (64.00, 93.00)	0.366
Total bilirubin (TBIL)		10.40 (8.00, 13.80)	10.40 (8.00, 13.50)	10.80 (8.00, 14.25)	0.801
Indirect bilirubin (IBIL)		7.70 (5.60, 10.50)	7.60 (5.60, 10.33)	7.80 (5.60, 10.60)	0.764
Total protein (TP)		73.40 (70.80, 75.95)	73.40 (70.90, 75.80)	73.40 (70.70, 76.25)	0.836
Albumin (ALB)		44.50 (43.00, 46.00)	44.60 (43.10, 46.10)	44.20 (42.75, 45.80)	0.173
Globulin (GLO)		25.60 (23.90, 28.15)	25.55 (23.90, 27.82)	25.90 (23.80, 28.30)	0.324
Albumin/Globulin ratio (A/G)		1.74 (1.58, 1.88)	1.74 (1.60, 1.91)	1.73 (1.56, 1.88)	0.218
Other biomarkers					
Homocysteine (Hcy)		12.55 (10.04, 16.04)	12.04 (9.87, 15.50)	13.63 (10.39, 16.86)	0.005

Categorical variables are presented as *n* (%). Continuous variables are presented as median (interquartile range). The low/medium-risk group was defined as having a China-PAR 10-year ASCVD risk score <10%, and the high-risk group was defined as having a score ≥10%. *P*-values were calculated using the chi-square test (for categorical variables) or the Mann–Whitney *U* test (for continuous variables). SBP, systolic blood pressure; DBP, diastolic blood pressure; TC, total cholesterol; HDL-C, high-density lipoprotein cholesterol; Lp(a), lipoprotein(a); sdLDL-C, small dense low-density lipoprotein cholesterol; HCY, homocysteine; hs-cTnI, high-sensitivity cardiac troponin I; NT-proBNP, N-terminal pro-B-type natriuretic peptide; TG, triglycerides; LDL-C, low-density lipoprotein cholesterol; FPG, fasting plasma glucose; UA, uric acid; WHR, waist-to-hip ratio; WBC, white blood cell count; NEUT%, neutrophil percentage; LYM%, lymphocyte percentage; NEUT_abs, neutrophil absolute count; MONO_abs, monocyte absolute count; RBC, red blood cell count; HGB, hemoglobin; HCT, hematocrit; MCV, mean corpuscular volume; MCH, mean corpuscular hemoglobin; MCHC, mean corpuscular hemoglobin concentration; RDW-SD, red cell distribution width-standard deviation; RDW-CV, red cell distribution width-coefficient of variation; PLT, platelet count; PCT, plateletcrit; MPV, mean platelet volume; PDW, platelet distribution width; P-LCR, platelet large cell ratio; CysC, cystatin C; AC, atherogenic coefficient; TBIL, total bilirubin; IBIL, indirect bilirubin; ALT, alanine aminotransferase; AST, aspartate aminotransferase; GGT, gamma-glutamyl transferase; ALP, alkaline phosphatase; TP, total protein; ALB, albumin; GLO, globulin; A/G ratio, albumin-to-globulin ratio.

### Correlation analysis between laboratory indicators and China-PAR risk scores

To systematically evaluate the strength of association between candidate indicators and the continuous China-PAR 10-year ASCVD risk score, Spearman correlation analysis was performed after excluding identifiers and outcome variables. FDR-adjusted *q* values were reported for the multiple biomarker correlations (Table 2). Figure 2 presents scatter plots of the 16 variables most strongly correlated with the risk score.

**Table 2:** Correlation analysis results between laboratory indicators and the China-PAR 10-year ASCVD risk score.

Variable	*N*	Spearman_rho	abs_rho	*P* value	FDR *q* value
FPG	511	0.4	0.4	<0.001	<0.001
CysC	511	0.395	0.395	<0.001	<0.001
hs_cTnI	511	0.293	0.293	<0.001	<0.001
AC	511	0.293	0.293	<0.001	<0.001
GGT	511	0.248	0.248	<0.001	<0.001
Creatinine	511	0.238	0.238	<0.001	<0.001
HCY	511	0.223	0.223	<0.001	<0.001
NEUT_abs	511	0.219	0.219	<0.001	<0.001
UA	511	0.217	0.217	<0.001	<0.001
LYM_pct	511	−0.213	0.213	<0.001	<0.001
NT_proBNP	511	0.204	0.204	<0.001	<0.001
NEUT_pct	511	0.189	0.189	<0.001	<0.001
PDW	511	0.186	0.186	<0.001	<0.001
MONO_abs	511	0.178	0.178	<0.001	<0.001
WBC	511	0.168	0.168	<0.001	<0.001
HGB	511	0.145	0.145	0.001	0.003
RBC	511	0.134	0.134	0.002	0.006
Albumin/Globulin ratio (A/G)	511	−0.129	0.129	0.003	0.008
GLO	511	0.127	0.127	0.004	0.009
HCT	511	0.126	0.126	0.004	0.009

This table presents the correlation analysis results of all tested laboratory indicators with the 10-year ASCVD risk score, sorted in descending order by the absolute correlation coefficient (|rho|). Spearman's rank correlation coefficient (rho) is reported for all continuous laboratory indicators (demographic features and categorical variables are excluded). The *P*-value is from a two-sided significance test. Variables with a *P* value <0.05 are highlighted in bold. FDR *q* value indicates the false discovery rate adjusted *P*-value.

**Figure 2:**
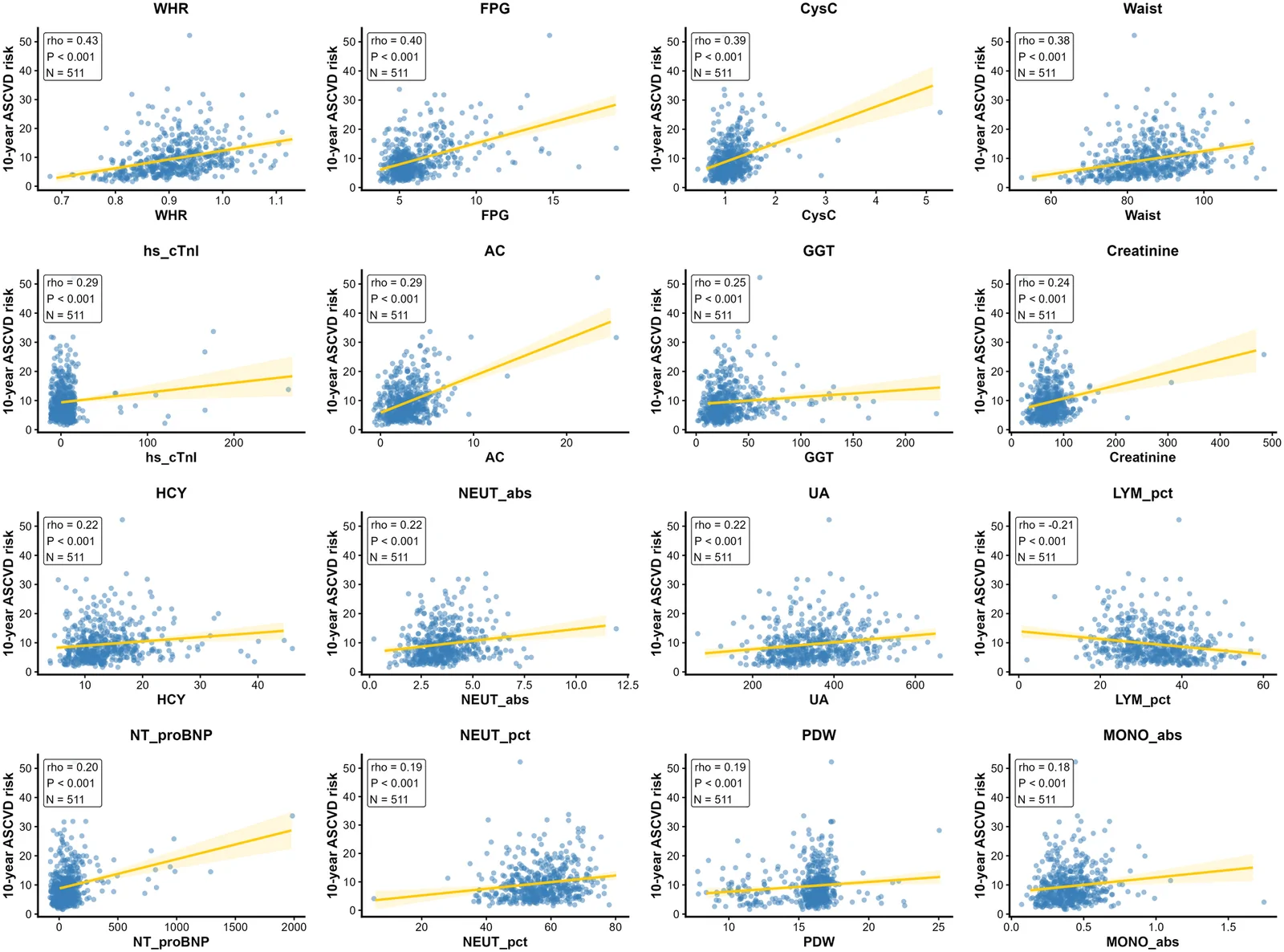
Scatter plots of laboratory indicators vs. China–PAR 10-year ASCVD risk scores (top 16 variables). This figure displays the 16 indicators most strongly correlated with the calculated China-PAR risk score. Each scatter plot illustrates the relationship between a single variable and the 10-year ASCVD risk score. Blue dots represent individual data points, the yellow trend line indicates the linear fit, and the light-yellow shaded area represents the 95% confidence interval. rho denotes the Spearman rank correlation coefficient, the *P*-value indicates the two-sided significance level of the correlation test, and *N* is the number of valid samples. WHR, waist-to-hip ratio; FPG, fasting plasma glucose; CysC, cystatin C; Waist, waist circumference; hs_cTnI, high-sensitivity cardiac troponin I; AC, atherogenic coefficient; GGT, gamma-glutamyl transferase; Creatinine, serum creatinine; HCY, homocysteine; NEUT_abs, neutrophil absolute count; UA, uric acid; LYM%, lymphocyte percentage; NT_proBNP, N-terminal pro-B-type natriuretic peptide; NEUT%, neutrophil percentage; PDW, platelet distribution width; MONO_abs, absolute monocyte count.

Among all candidate indicators, the WHR showed the strongest correlation with the risk score (rho = 0.43, *P* < 0.001), followed by FPG (rho = 0.40, *P* < 0.001) and CysC (rho = 0.39, *P* < 0.001). The anthropometric measure waist circumference also demonstrated a strong positive correlation (rho = 0.38, *P* < 0.001).

Among novel cardiovascular markers, hs-cTnI and the AC showed comparable strengths of association (both rho = 0.29, *P* < 0.001). NT-proBNP (rho = 0.20, *P* < 0.001) and HCY (rho = 0.22, *P* < 0.001) were also significantly correlated with the risk score, while sdLDL-C exhibited a relatively weaker correlation (rho = 0.14, *P* = 0.001). Notably, Lp(a) showed a weak negative correlation with the risk score (rho = −0.09, *P* = 0.033).

All renal function-related indicators were significantly correlated with the risk score. In addition to CysC (rho = 0.39), serum creatinine (rho = 0.24, *P* < 0.001) and UA (rho = 0.22, *P* < 0.001) also demonstrated significant associations. Among liver function indicators, GGT showed a significant positive correlation with the risk score (rho = 0.25, *P* < 0.001).

Regarding inflammation-related complete blood count parameters, neutrophil absolute count (NEUT_abs, rho = 0.22, *P* < 0.001), neutrophil percentage (NEUT%), rho = 0.19, *P* < 0.001), and MONO absolute count (MONO_abs, rho = 0.18, *P* < 0.001) were positively correlated with the risk score. In contrast, LYM percentage showed a negative correlation (LYM%, rho = −0.21, *P* < 0.001). PDW also exhibited a significant positive correlation (rho = 0.19, *P* < 0.001). WBC showed a relatively weaker correlation with the risk score (rho = 0.17, *P* < 0.001).

Notably, LDL-C, although a classic risk factor for atherosclerosis, showed no significant correlation with the China-PAR risk score (rho = 0.05, *P* = 0.252). Some liver function indicators, such as ALT (rho = 0.02, *P* = 0.584) and AST (rho = 0.001, *P* = 0.987), were not significantly associated with the risk score.

### Exploratory LASSO feature selection for China-PAR-defined high-risk status

To identify a parsimonious set of laboratory features associated with the calculated high-risk category, exploratory LASSO logistic regression was performed among candidate laboratory variables after excluding variables directly used in the China-PAR equation and identifying information. The binary outcome was China-PAR ≥10% vs. <10%; therefore, this analysis assessed discrimination of a score-derived risk category rather than prediction of clinical ASCVD events.

The penalty parameter was optimized via 10-fold cross-validation. The cross-validation curve showed an AUC of 0.824 at lambda.min and 0.794 at lambda.1se (Figure 3A). At lambda.min, four nonzero features were retained: CysC, FPG, AC, and NT-proBNP, whereas the more parsimonious lambda.1se solution retained CysC and FPG (Figure 3B). Selected coefficients and the complete 47-variable candidate list are provided in Supplementary Table S1. The cross-validated AUC reflects discrimination of China-PAR-defined high-risk status and should not be interpreted as incremental prognostic performance beyond China-PAR.

**Figure 3:**
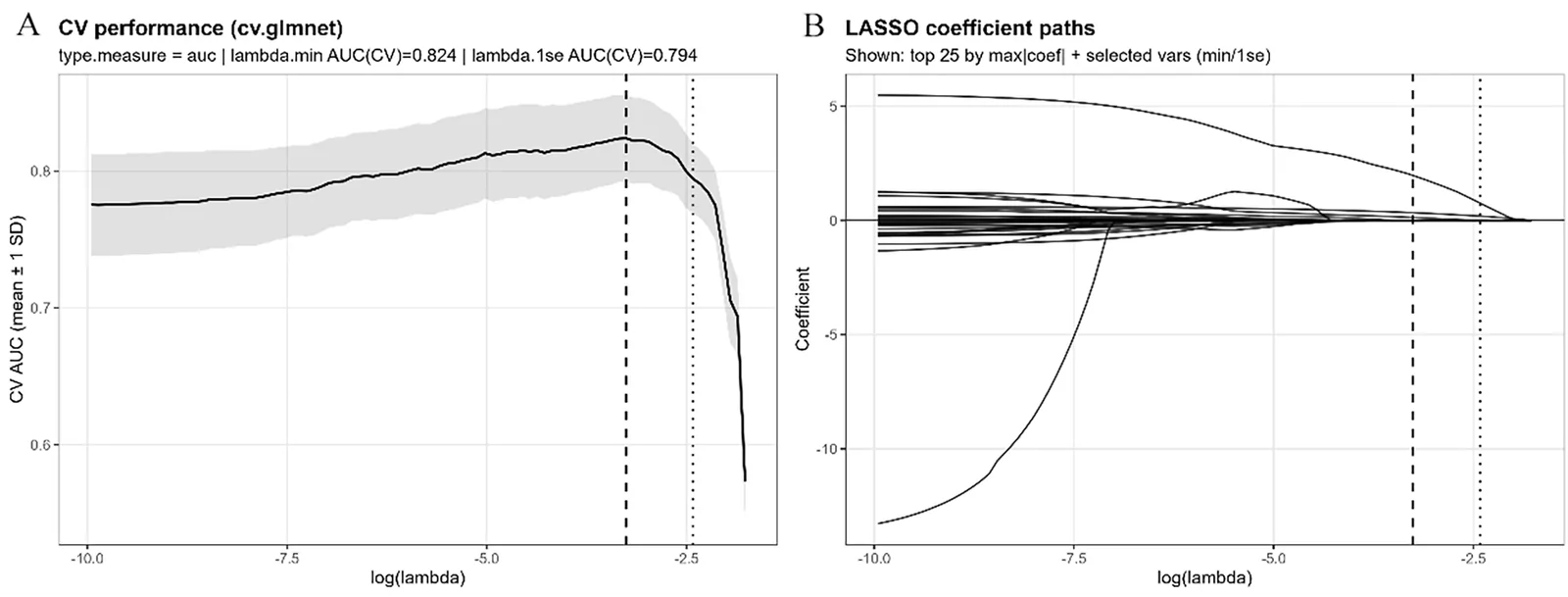
Results of feature selection by LASSO regression. **(A)** Ten-fold cross-validation curve: The *x*-axis represents the penalty parameter on a logarithmic scale [log(*λ*)], and the *y*-axis represents the mean cross-validated AUC with its standard deviation. The dashed vertical line marks lambda.min, at which the cross-validated AUC was 0.824, and the dotted vertical line marks lambda.1se, at which the AUC was 0.794. These AUC values quantify discrimination of the China-PAR-defined high-risk category and do not represent prediction of observed ASCVD events. **(B)** LASSO coefficient path plot: This plot shows the trajectories of coefficients for the top 25 variables as log(*λ*) changes. The *x*-axis is log(*λ*), and the *y*-axis is the coefficient. The dashed vertical line indicates lambda.min and the dotted line indicates lambda.1se. At lambda.min, four nonzero features were retained: CysC, FPG, AC, and NT-proBNP; at lambda.1se, CysC and FPG remained nonzero.

The coefficient path plot showed progressive shrinkage of coefficients as the penalty increased. In 1,000 successful bootstrap resamples, the four features retained at lambda.min had the highest selection frequencies among the candidate variables: CysC, 100.0%; FPG, 100.0%; AC, 99.8%; and NT-proBNP, 97.4% (Supplementary Table S1). These selected features differ in their relationship to the China-PAR equation: FPG is clinically linked to diabetes status, and AC is mathematically derived from TC and HDL-C, whereas CysC and NT-proBNP are not components of the China-PAR model. This distinction was carried forward into the interpretation of the results.

### Logistic associations of the selected biomarkers with high-risk status

To facilitate comparison of effect sizes, the four LASSO-selected variables were standardized using *z*-score transformation and included in logistic regression analyses. NT-proBNP was natural-log transformed before standardization. Table 3 presents the associations with China-PAR-defined high-risk status (≥10%). In univariate analyses, all four biomarkers were significantly associated with high-risk status (all *P* < 0.001). The ORs represent a one-standard-deviation increase in each biomarker. The corresponding pre-standardization mean ± SD values were FPG, 6.11 ± 1.84 mmol/L; CysC, 1.12 ± 0.30 mg/L; AC, 2.97 ± 2.06; and log_NT_proBNP, 3.84 ± 0.89. The univariate ORs were 2.875 (95% CI: 2.089–3.958) for CysC, 2.636 (95% CI: 2.005–3.465) for FPG, 2.054 (95% CI: 1.570–2.688) for AC, and 1.728 (95% CI: 1.416–2.107) for log_NT_proBNP.

**Table 3:** Univariate and multivariate logistic regression analysis of LASSO-selected biomarkers associated with China-PAR-defined high-risk status.

Variable	Univariate analysis		Multivariate analysis	
	OR (95% CI)	*P* value	aOR (95% CI)	*P* value
z_FPG	2.636 (2.005, 3.465)	<0.001	2.772 (2.047, 3.753)	<0.001
z_CysC	2.875 (2.089, 3.958)	<0.001	2.731 (1.881, 3.964)	<0.001
z_AC	2.054 (1.57, 2.688)	<0.001	2.085 (1.524, 2.852)	<0.001
z_logNTproBNP	1.728 (1.416, 2.107)	<0.001	1.582 (1.243, 2.015)	<0.001

This table presents the results of univariate and multivariate logistic regression analyses of associations with China-PAR-defined high-risk status (score ≥10%) using the four LASSO-selected biomarkers after *z*-score standardization. In the univariate analysis, each biomarker was entered individually. In the multivariate analysis, all four biomarkers were included simultaneously and mutually adjusted for one another. The odds ratio (OR) or adjusted odds ratio (aOR) represents the change in odds of high-risk status associated with a one-standard-deviation increase in the biomarker level. NT-proBNP was log-transformed before standardization. 95% CI, 95% confidence interval. *P*-values are from the two-sided Wald test. The mean ± standard deviation of variables before standardization were: FPG, 6.11 ± 1.84 mmol/L; CysC, 1.12 ± 0.30 mg/L; AC, 2.97 ± 2.06; log_NT_proBNP, 3.84 ± 0.89. z_FPG, standardized fasting plasma glucose; z_CysC, standardized cystatin C; z_AC, standardized atherogenic coefficient; z_logNTproBNP, standardized logarithmically transformed NT-proBNP; OR, odds ratio; aOR, adjusted odds ratio; CI, confidence interval.

In the mutually adjusted logistic regression model, all four biomarkers remained associated with China-PAR-defined high-risk status (all *P* < 0.001). The aOR was 2.772 for FPG (95% CI: 2.047–3.753), 2.731 for CysC (95% CI: 1.881–3.964), 2.085 for AC (95% CI: 1.524–2.852), and 1.582 for log_NT_proBNP (95% CI: 1.243–2.015). These estimates quantify associations with the formula-derived high-risk category after mutual adjustment among the four selected biomarkers; they do not establish incremental predictive value beyond China-PAR or prediction of future ASCVD events.

### Quartile stratification of the selected biomarkers

To describe graded associations between the selected biomarkers and estimated China-PAR risk, the study population was divided into quartile groups (Q1–Q4) according to each biomarker level. The mean calculated risk score and the distribution of the China-PAR-defined high-risk proportion were summarized across quartiles (Figure 4). Exact quartile cutoffs, sample sizes, risk-score summaries, high-risk proportions with 95% confidence intervals, and formal trend statistics are provided in Supplementary Table S2.

**Figure 4:**
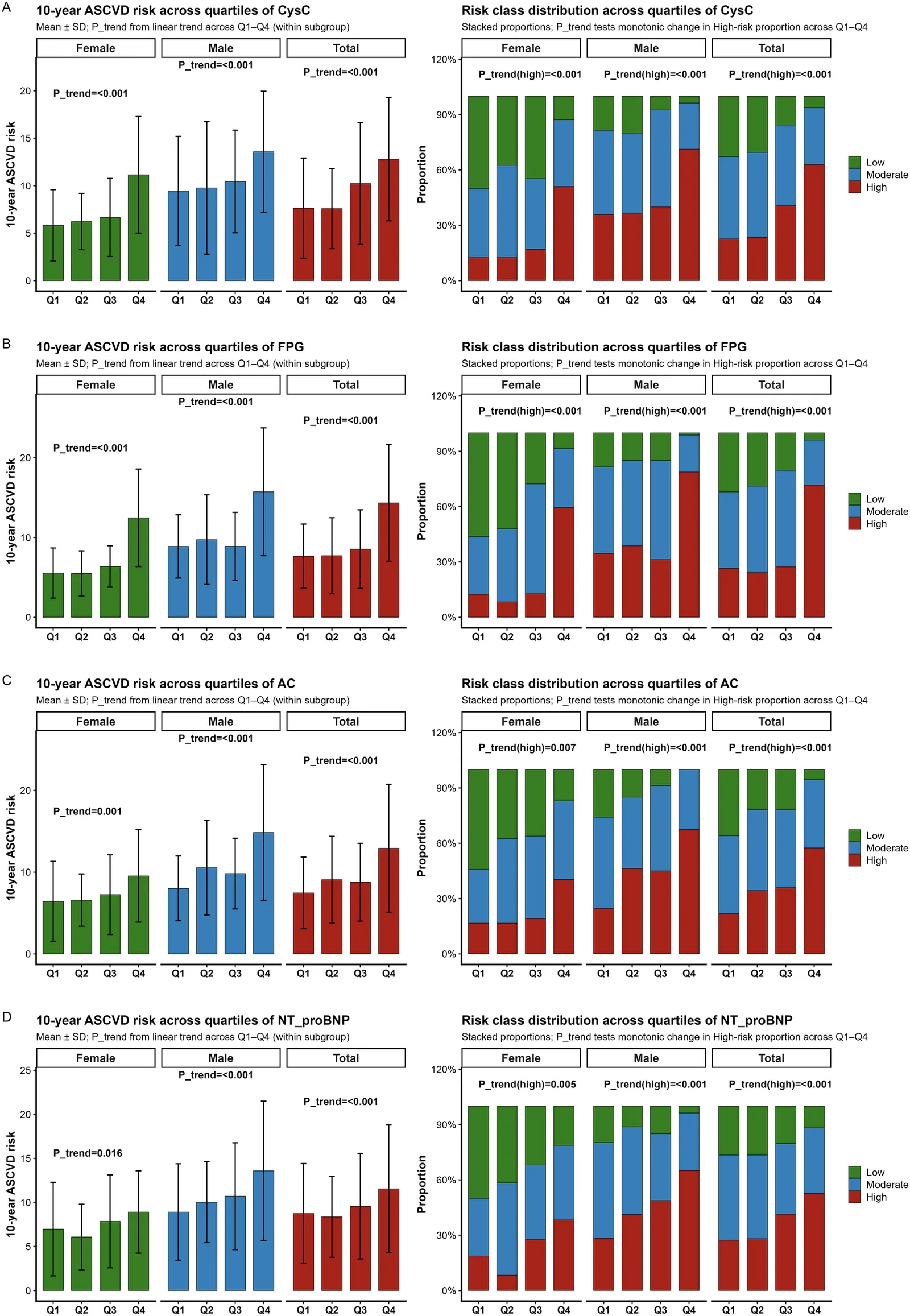
Distribution of calculated 10-year ASCVD risk scores and China–PAR, defined higH–risk proportions by quartile stratification of the four LASSO–selected biomarkers. **(A)** Cystatin C (CysC) stratification; **(B)** Fasting plasma glucose (FPG) stratification; **(C)** Atherogenic coefficient (AC) stratification; **(D)** N-terminal pro-B-type natriuretic peptide (NT-proBNP) stratification. The left bar charts show the mean ± SD of the calculated 10-year ASCVD risk score within each quartile group. *P* for trend indicates the *P*-value for the linear trend test. The right stacked bar charts show the proportional distribution of China-PAR risk categories (low, intermediate, and high) across quartiles, and *P* for trend (high-risk proportion) indicates the *P*-value for the monotonic trend in the proportion classified as high risk. Sex-stratified trend tests are descriptive and do not represent formal biomarker-by-sex interaction tests.

CysC Stratification: As the CysC quartile increased, the mean 10-year ASCVD risk score showed a significant ascending trend (*P* for trend <0.001). In the overall population, the risk score increased from approximately 5% in Q1 to about 12% in Q4. The proportion of high-risk (≥10%) individuals also demonstrated a significant monotonic increase across quartiles (*P* for trend < 0.001), rising from about 15% in Q1 to approximately 60% in Q4. Sex-stratified analysis revealed consistent trends in both female and male subgroups (both *P* for trend < 0.001) (Figure 4A).

FPG Stratification: FPG quartiles showed a significant positive trend with the risk score (overall population *P* for trend < 0.001). In the female subgroup, the score increased from about 3% in Q1 to about 12% in Q4 (*P* for trend < 0.001), while in the male subgroup, it increased from about 8% in Q1 to about 15% in Q4 (*P* for trend < 0.001). The high-risk proportion also increased significantly across quartiles (overall population *P* for trend < 0.001), approaching 70% in Q4 compared to only about 15% in Q1 (Figure 4B).

AC Stratification: Increasing AC quartiles were associated with higher calculated risk scores (overall population *P* for trend < 0.001). Significant within-sex trends were observed in both males (*P* for trend < 0.001) and females (*P* for trend = 0.001). The high-risk proportion also increased across AC quartiles (overall population *P* for trend < 0.001), with approximately 60% in Q4 compared with about 20% in Q1; the within-female trend for the high-risk proportion was *P* for trend = 0.007 (Figure 4C). These within-sex *P* values were treated descriptively and were not used to infer a sex interaction.

NT-proBNP Stratification: NT-proBNP quartiles showed a positive trend with the calculated risk score (overall population *P* for trend < 0.001). Significant within-sex trends were observed in males (*P* for trend < 0.001) and females (*P* for trend = 0.016). The high-risk proportion also increased across quartiles in the overall population (*P* for trend < 0.001), with within-sex trends in males (*P* for trend < 0.001) and females (*P* for trend = 0.005) (Figure 4D). These subgroup trend tests were descriptive and did not constitute formal biomarker-by-sex interaction tests.

In summary, the quartile stratification analysis demonstrated graded associations between all four selected biomarkers and the calculated China-PAR risk score, with generally increasing high-risk proportions across quartiles. Positive trends were observed within both male and female subgroups; however, the sex-stratified analyses were descriptive and no between-sex difference was inferred from differences in subgroup *P* values.

## Discussion

This study applied the China-PAR equation in 511 adults aged ≥60 years undergoing routine health examinations and characterized cross-sectional associations between cardiometabolic biomarkers and the calculated 10-year ASCVD risk score. Overall, 37.4% of participants were classified as high risk (≥10%). Exploratory LASSO feature selection retained CysC, FPG, AC, and NT-proBNP as features associated with China-PAR-defined high-risk status. These results describe biomarker patterns accompanying higher estimated China-PAR risk and should not be interpreted as prediction of incident ASCVD events.

### Current application status of the China-PAR model

The China-PAR equation was developed by Yang et al. in 2016 based on 106,281 participants and demonstrated good discriminative ability in the development cohort (C-statistic 0.794) (5). Compared with risk equations derived primarily from Western populations, China-PAR was specifically developed for Chinese adults and has subsequently been evaluated in additional Chinese cohorts (7, 15–17). The finding that 37.4% of the present older health examination cohort met the ≥10% high-risk threshold therefore describes the distribution of estimated risk in this specific setting. Examining biomarkers alongside China-PAR may help characterize phenotypic heterogeneity across calculated risk strata, but this cross-sectional design does not establish incremental prognostic value beyond the China-PAR equation.

### Associations of selected biomarkers with estimated China-PAR risk

#### Association of CysC with estimated risk

CysC has established associations with cardiovascular outcomes in prospective cohorts. Shlipak et al. reported that higher CysC levels were associated with cardiovascular death and other cardiovascular outcomes in community-dwelling older adults (15, 16), and a subsequent meta-analysis also supported associations with long-term mortality (17). In the present cross-sectional analysis, CysC showed a strong correlation with the calculated China-PAR score (rho = 0.39), and its mutually adjusted OR for China-PAR-defined high-risk status was 2.731 per SD. Because CysC is not included in the China-PAR equation, this association is not produced by direct mathematical reuse of a score component. Nevertheless, the present study cannot determine whether CysC adds prognostic information for future ASCVD events.

#### Association of FPG with estimated risk

Prospective evidence supports a relationship between dysglycemia and vascular risk. A large meta-analysis found higher vascular risk across increasing fasting glucose levels (18), and a Korean cohort also demonstrated a nonlinear association between fasting glucose and cardiovascular outcomes (19). In the present study, FPG showed a strong correlation with the calculated China-PAR score (rho = 0.40) and an aOR of 2.772 per SD for China-PAR-defined high-risk status. However, diabetes status is itself a China-PAR component, and FPG contributes to the clinical definition of diabetes in this cohort. Therefore, part of the observed FPG association is expected from clinical coupling with the score and should not be interpreted as independent information beyond China-PAR.

#### Association of the atherogenic coefficient with estimated risk

The lipid variable analyzed in this study is the atherogenic coefficient (AC), calculated as (TC - HDL-C)/HDL-C, which is equivalent to the non-HDL-C/HDL-C ratio. This measure is distinct from the atherogenic index of plasma, which is defined as log(TG/HDL-C). These terms and formulas should not be treated as interchangeable (20–22). In the present study, AC correlated with the calculated China-PAR score (rho = 0.29) and was associated with China-PAR-defined high-risk status after mutual adjustment (aOR = 2.085 per SD). Because AC is calculated directly from TC and HDL-C, both of which are components of the China-PAR equation, this finding is mathematically coupled to score construction and should not be interpreted as independent biological or prognostic information beyond China-PAR.

#### Association of NT-proBNP with estimated risk

NT-proBNP has been associated with cardiovascular outcomes in multiple prospective cohorts. The Framingham study reported associations between natriuretic peptide levels and cardiovascular events and death (23), and later studies also linked higher NT-proBNP levels with cardiovascular and all-cause mortality (24, 25). In the present study, NT-proBNP showed a more modest correlation with the calculated China-PAR score (rho = 0.20) but remained associated with China-PAR-defined high-risk status after mutual adjustment (aOR = 1.582 per SD for log-transformed NT-proBNP). NT-proBNP is not a component of the China-PAR equation, making it a non-overlapping biomarker signal in this cross-sectional analysis; however, its incremental prognostic value cannot be established without observed cardiovascular outcomes.

#### Key findings of this study

The correlation analysis showed that WHR had the strongest association with the calculated China-PAR risk score (rho = 0.43), followed by FPG and CysC. Interpretation of these findings requires attention to overlap with score construction. WHR is closely related to waist circumference, a China-PAR component; FPG is clinically linked to diabetes status; and AC is derived from TC and HDL-C. In contrast, CysC and NT-proBNP are not variables in the China-PAR equation. Accordingly, the four LASSO-selected biomarkers can be separated into overlapping features (FPG and AC) and non-overlapping features (CysC and NT-proBNP). The latter may be of particular interest for future prospective evaluation, but the current data do not demonstrate incremental prediction beyond China-PAR.

Quartile stratification showed graded increases in calculated China-PAR risk and in the proportion classified as high risk across CysC, FPG, AC, and NT-proBNP quartiles. Similar directional trends were observed in both sex subgroups. Because no formal biomarker-by-sex interaction test was performed, differences in within-sex *P* values were not interpreted as evidence of sex-specific effects, and biological explanations for between-sex differences were not inferred from these analyses.

#### Clinical implications and future prospects

In routine health examinations, CysC, FPG, AC, and NT-proBNP may provide clinically interpretable context for higher estimated China-PAR risk by highlighting accompanying metabolic, renal, lipid, and cardiac abnormalities. Although these cross-sectional findings do not establish incremental prediction beyond China-PAR or support formal risk reclassification, they may increase clinical awareness of individuals with unfavorable biomarker profiles and prompt closer assessment of modifiable cardiovascular risk factors and relevant organ status. FPG and AC partly reflect variables already represented in China-PAR, whereas CysC and NT-proBNP may draw attention to renal and cardiac features not directly captured by the equation. Prospective studies with observed ASCVD events are required before any formal incremental risk-stratification role can be established.

#### Study limitations

This study has several limitations. First, the cross-sectional outcome was an estimated China-PAR risk score rather than an observed cardiovascular event; therefore, the analyses establish association with a calculated score and cannot demonstrate causality, prognosis, or incremental clinical prediction. Second, mathematical and clinical coupling is unavoidable for several variables: AC is derived from TC and HDL-C, FPG is closely linked to diabetes status, and WHR is related to waist circumference, all of which overlap with variables used in China-PAR. These associations should therefore be interpreted more cautiously than those for non-overlapping biomarkers such as CysC and NT-proBNP. Third, the cross-validated LASSO AUC quantifies discrimination of a formula-derived high-risk category and does not represent improvement in prediction beyond China-PAR; metrics such as reclassification, calibration against observed outcomes, and decision-curve analysis require prospective event data. Fourth, participants were recruited from a single health examination center over a relatively short period, 98.6% were from Southern China, and all were aged ≥60 years. Individuals who attend routine health examinations may also differ from the general older population in health awareness, socioeconomic characteristics, or baseline health status, introducing potential selection bias; complete-case exclusion of 55 of 566 initially assessed individuals may further contribute to selection. Fifth, sex-stratified analyses were descriptive and no formal interaction tests were performed. Finally, factors affecting NT-proBNP and other laboratory biomarkers could not be comprehensively accounted for in this cross-sectional dataset. These considerations limit generalizability and support prospective validation in broader populations with adjudicated clinical outcomes.

## Conclusion

In summary, this cross-sectional study characterized cardiometabolic biomarker profiles associated with estimated 10-year ASCVD risk calculated by China-PAR in an older adult health examination cohort. CysC, FPG, AC, and NT-proBNP were retained by exploratory LASSO feature selection and were associated with China-PAR-defined high-risk status. FPG, AC, and WHR overlap clinically or mathematically with components of the China-PAR equation and should therefore be interpreted partly as coupled signals of score construction. CysC and NT-proBNP are not China-PAR components and represent non-overlapping associations that merit prospective study. Although the present findings do not establish prediction of future ASCVD events or incremental performance beyond China-PAR, the observed biomarker profiles may help increase clinical awareness of individuals with higher estimated cardiovascular risk during routine health examinations and support more comprehensive risk-factor assessment. Prospective longitudinal studies with observed cardiovascular outcomes are needed to determine their prognostic significance.

## Data Availability

The datasets generated and analysed during the current study are not publicly available due to patient privacy restrictions, but are available from the corresponding author on reasonable request.

## References

[1] Roth GA Mensah GA Johnson CO Addolorato G Ammirati E Baddour LM. Global burden of cardiovascular diseases and risk factors, 1990–2019: update from the GBD 2019 study. J Am Coll Cardiol. (2020) 76(25):2982–3021. 10.1016/j.jacc.2020.11.010 33309175 PMC7755038

[2] Vogel B Acevedo M Appelman Y Bairey Merz CN Chieffo A Figtree GA. The lancet women and cardiovascular disease commission: reducing the global burden by 2030. Lancet. (2021) 397(10292):2385–438. 10.1016/S0140-6736(21)00684-X 34010613

[3] Liu J. Predictive value for the Chinese population of the framingham CHD risk assessment tool compared with the Chinese multi-provincial cohort study. J Am Med Assoc. (2004) 291(21):2591–9. 10.1001/jama.291.21.2591 15173150

[4] Zhang M. Prediction of 10-year atherosclerotic cardiovascular disease risk among adults aged 40–79 years in China: a nationally representative survey. Biomed Environ Sci. (2017) 30(4):244–54. 10.3967/bes2017.034 28494834

[5] Yang X Li J Hu D Chen J Li Y Huang J. Predicting the 10-year risks of atherosclerotic cardiovascular disease in Chinese population: the China-PAR project (prediction for ASCVD risk in China). Circulation. (2016) 134(19):1430–40. 10.1161/CIRCULATIONAHA.116.022367 27682885

[6] Gutierrez JA Aday AW Patel MR Jones WS. Polyvascular disease: reappraisal of the current clinical landscape. Circ Cardiovasc Interv. (2019) 12(12):e007385. 10.1161/CIRCINTERVENTIONS.119.007385 31833412 PMC7660526

[7] Liu X Shen P Zhang D Sun Y Chen Y Liang J. Evaluation of atherosclerotic cardiovascular risk prediction models in China: results from the CHERRY study. JACC Asia. (2022) 2(1):33–43. 10.1016/j.jacasi.2021.10.007 36340248 PMC9627894

[8] Naghavi M Libby P Falk E Casscells SW Litovsky S Rumberger J. From vulnerable plaque to vulnerable patient: a call for new definitions and risk assessment strategies: part I. Circulation. (2003) 108(14):1664–72. 10.1161/01.CIR.0000087480.94275.97 14530185

[9] Wang TJ. Assessing the role of circulating, genetic, and imaging biomarkers in cardiovascular risk prediction. Circulation. (2011) 123(5):551–65. 10.1161/CIRCULATIONAHA.109.912568 21300963 PMC3059807

[10] de Boer RA Nayor M Defilippi CR Enserro D Bhambhani V Kizer JR. Association of cardiovascular biomarkers with incident heart failure with preserved and reduced ejection fraction. JAMA Cardiol. (2018) 3(3):215–24. 10.1001/jamacardio.2017.4987 29322198 PMC5862778

[11] Lindholm D Lindbäck J Armstrong PW Budaj A Cannon CP Granger CB. Biomarker-based risk model to predict cardiovascular mortality in patients with stable coronary disease. J Am Coll Cardiol. (2017) 70(7):813–26. 10.1016/j.jacc.2017.06.030 28797349

[12] Brouwers FP Van Gilst WH Damman K Van Den Berg MP Gansevoort RT Bakker SJL. Clinical risk stratification optimizes value of biomarkers to predict new-onset heart failure in a community-based cohort. Circ Heart Fail. (2014) 7(5):723–31. 10.1161/CIRCHEARTFAILURE.114.001185 25057112

[13] Chinese Society of Cardiology of Chinese Medical Association, Cardiovascular Disease Prevention and Rehabilitation Committee of Chinese Association of Rehabilitation Medicine, Cardiovascular Disease Committee of Chinese Association of Gerontology and Geriatrics, Thrombosis Prevention and Treatment Committee of Chinese Medical Doctor Association. Chinese guideline on the primary prevention of cardiovascular diseases. Zhonghua Xin Xue Guan Bing Za Zhi. (2020) 48(12):1000–38. 10.3760/cma.j.cn112148-20201009-00796 33355747

[14] Li HH. Applying the China-PAR risk algorithm to assess 10-year atherosclerotic cardiovascular disease risk in populations receiving routine physical examinations in eastern China. Biomed Environ Sci. (2019) 32(2):87–95. 10.3967/bes2019.014 30862340

[15] Shlipak MG Sarnak MJ Katz R Fried LF Seliger SL Newman AB. Cystatin C and the risk of death and cardiovascular events among elderly persons. N Engl J Med. (2005) 352(20):2049–60. 10.1056/NEJMoa043161 15901858

[16] Shlipak MG Katz R Sarnak MJ Fried LF Newman AB Stehman-Breen C. Cystatin C and prognosis for cardiovascular and kidney outcomes in elderly persons without chronic kidney disease. Ann Intern Med. (2006) 145(4):237–46. 10.7326/0003-4819-145-4-200608150-00003 16908914

[17] Tanto C Bawazier LA Marbun MBH Rizka A Renaldi K. Cystatin C as predictor of long-term mortality in elderly: a systematic review and meta-analysis. SN Compr Clin Med. (2022) 4(1):171. 10.1007/s42399-022-01233-x

[18] Sarwar N. Diabetes mellitus, fasting blood glucose concentration, and risk of vascular disease: a collaborative meta-analysis of 102 prospective studies. Lancet. (2010) 375(9733):2215–22. 10.1016/S0140-6736(10)60484-9 20609967 PMC2904878

[19] Park C Guallar E Linton JA Lee D-C Jang Y Son DK. Fasting glucose level and the risk of incident atherosclerotic cardiovascular diseases. Diabetes Care. (2013) 36(7):1988–93. 10.2337/dc12-1577 23404299 PMC3687304

[20] Dobiásová M Frohlich J. The plasma parameter log (TG/HDL-C) as an atherogenic index: correlation with lipoprotein particle size and esterification rate in apoB-lipoprotein-depleted plasma [FER(HDL)]. Clin Biochem. (2001) 34(7):583–8. 10.1016/S0009-9120(01)00263-6 11738396

[21] Sujatha R Kavitha S. Atherogenic indices in stroke patients: a retrospective study. Iran J Neurol. (2017) 16(2):78–82. PMID: 28761629 28761629 PMC5526781

[22] Nam BH Kannel WB D'Agostino RB. Search for an optimal atherogenic lipid risk profile: from the Framingham study. Am J Cardiol. (2006) 97(3):372–5. 10.1016/j.amjcard.2005.08.055 16442398

[23] Wang TJ Larson MG Levy D Benjamin EJ Leip EP Omland T. Plasma natriuretic peptide levels and the risk of cardiovascular events and death. N Engl J Med. (2004) 350(7):655–63. 10.1056/NEJMoa031994 14960742

[24] Rudolf H Mügge A Trampisch HJ Scharnagl H März W Kara K. NT-proBNP for risk prediction of cardiovascular events and all-cause mortality: the getABI-study. Int J Cardiol Heart Vasc. (2020) 29:100553. 10.1016/j.ijcha.2020.100553 32529024 PMC7280763

[25] Tcheugui JB Zhang S McEvoy JW Ndumele CE Hoogeveen RC Coresh J. Elevated NT-ProBNP as a cardiovascular disease risk equivalent: evidence from the atherosclerosis risk in communities (ARIC) study. Am J Med. (2022) 135(12):1461–7. 10.1016/j.amjmed.2022.07.012 36007589 PMC10208080

