# Supplementary material for: Cardiometabolic biomarker profiles associated with estimated 10-year ASCVD risk by the China-PAR model in an older adult health examination cohort: a cross-sectional study
Source: Front Med (Lausanne). 2026 Oct 14;13:1897876. doi: 10.3389/fmed.2026.1897876 (PMC13713855; doi:10.3389/fmed.2026.1897876)
Supplement: Supplementary file 1 [file Table1.docx]

**Supplementary Table S1. LASSO candidate variables, selected coefficients, and bootstrap selection stability**

**Model summary**

| Analytic N | 511 | High-risk cases | 191 | Candidate variables | 47 |
| --- | --- | --- | --- | --- | --- |
| lambda.min | 0.038566 | Nonzero at lambda.min | 4 | CV AUC at lambda.min | 0.824 |
| lambda.1se | 0.089092 | Nonzero at lambda.1se | 2 | CV AUC at lambda.1se | 0.794 |

| Candidate variable | Coefficient at lambda.min | Coefficient at lambda.1se | Selected at lambda.min | Selected at lambda.1se | Bootstrap selected, n/1000 | Selection frequency (%) |
| --- | --- | --- | --- | --- | --- | --- |
| FPG | 0.341003 | 0.194093 | Yes | Yes | 1000/1000 | 100.0 |
| CysC | 1.968495 | 0.762367 | Yes | Yes | 1000/1000 | 100.0 |
| AC | 0.129916 | 0 | Yes | No | 998/1000 | 99.8 |
| NT-proBNP | 0.000641 | 0 | Yes | No | 974/1000 | 97.4 |
| ALP | 0 | 0 | No | No | 811/1000 | 81.1 |
| Urea | 0 | 0 | No | No | 808/1000 | 80.8 |
| PDW | 0 | 0 | No | No | 782/1000 | 78.2 |
| MCHC | 0 | 0 | No | No | 738/1000 | 73.8 |
| ALT | 0 | 0 | No | No | 729/1000 | 72.9 |
| PLT | 0 | 0 | No | No | 728/1000 | 72.8 |
| sdLDL-C | 0 | 0 | No | No | 672/1000 | 67.2 |
| RBC | 0 | 0 | No | No | 660/1000 | 66.0 |
| Hcy | 0 | 0 | No | No | 650/1000 | 65.0 |
| Lymphocyte count | 0 | 0 | No | No | 650/1000 | 65.0 |
| Lp(a) | 0 | 0 | No | No | 640/1000 | 64.0 |
| UA | 0 | 0 | No | No | 640/1000 | 64.0 |
| LDL-C | 0 | 0 | No | No | 631/1000 | 63.1 |
| GGT | 0 | 0 | No | No | 624/1000 | 62.4 |
| hs-cTnI | 0 | 0 | No | No | 606/1000 | 60.6 |
| AST | 0 | 0 | No | No | 603/1000 | 60.3 |
| MPV | 0 | 0 | No | No | 584/1000 | 58.4 |
| Monocytes (%) | 0 | 0 | No | No | 582/1000 | 58.2 |
| Basophils (%) | 0 | 0 | No | No | 577/1000 | 57.7 |
| TBIL | 0 | 0 | No | No | 570/1000 | 57.0 |
| TG | 0 | 0 | No | No | 562/1000 | 56.2 |
| Monocyte count | 0 | 0 | No | No | 561/1000 | 56.1 |
| Serum creatinine | 0 | 0 | No | No | 557/1000 | 55.7 |
| MCV | 0 | 0 | No | No | 552/1000 | 55.2 |
| ALB | 0 | 0 | No | No | 531/1000 | 53.1 |
| MID cell count | 0 | 0 | No | No | 529/1000 | 52.9 |
| GLO | 0 | 0 | No | No | 492/1000 | 49.2 |
| IBIL | 0 | 0 | No | No | 462/1000 | 46.2 |
| Basophil count | 0 | 0 | No | No | 460/1000 | 46.0 |
| RDW-CV | 0 | 0 | No | No | 455/1000 | 45.5 |
| Lymphocytes (%) | 0 | 0 | No | No | 435/1000 | 43.5 |
| RDW-SD | 0 | 0 | No | No | 435/1000 | 43.5 |
| A/G ratio | 0 | 0 | No | No | 431/1000 | 43.1 |
| Eosinophil count | 0 | 0 | No | No | 425/1000 | 42.5 |
| P-LCR | 0 | 0 | No | No | 417/1000 | 41.7 |
| Eosinophils (%) | 0 | 0 | No | No | 402/1000 | 40.2 |
| MCH | 0 | 0 | No | No | 360/1000 | 36.0 |
| Neutrophils (%) | 0 | 0 | No | No | 353/1000 | 35.3 |
| PCT | 0 | 0 | No | No | 344/1000 | 34.4 |
| TP | 0 | 0 | No | No | 322/1000 | 32.2 |
| HGB | 0 | 0 | No | No | 278/1000 | 27.8 |
| HCT | 0 | 0 | No | No | 239/1000 | 23.9 |
| WBC | 0 | 0 | No | No | 84/1000 | 8.4 |

**Note:** Candidate laboratory variables were entered into exploratory binomial LASSO after excluding identifiers and variables used directly in the China-PAR equation. The main-sample model used 10-fold cross-validation with AUC as the performance measure; lambda.min was used for primary feature selection and lambda.1se was examined as a more parsimonious reference. Bootstrap stability was assessed in 1,000 resamples; in each resample, cross-validated LASSO was refitted and variables selected at lambda.min were recorded. The coefficients shown are from the original-sample LASSO fit; zero indicates that the variable was not retained at the specified lambda. AC corresponds to the source-data variable previously labeled AI and is defined as (TC − HDL-C)/HDL-C. AUC, area under the receiver operating characteristic curve; AC, atherogenic coefficient; LASSO, least absolute shrinkage and selection operator.

**Supplementary Table S2. Quartile cutoffs and distribution of China-PAR risk across quartiles of the four LASSO-selected biomarkers**

**Panel A. Quartile cutoffs**

| Biomarker | Group | N | P25 | P50 | P75 |
| --- | --- | --- | --- | --- | --- |
| CysC (mg/L) | Total | 511 | 0.96 | 1.07 | 1.20 |
|  | Male | 321 | 1.00 | 1.10 | 1.23 |
|  | Female | 190 | 0.92 | 1.02 | 1.14 |
| FPG (mmol/L) | Total | 511 | 5.03 | 5.52 | 6.545 |
|  | Male | 321 | 5.03 | 5.65 | 6.70 |
|  | Female | 190 | 5.022 | 5.44 | 6.143 |
| AC | Total | 511 | 1.83 | 2.75 | 3.795 |
|  | Male | 321 | 1.82 | 2.71 | 3.86 |
|  | Female | 190 | 1.845 | 2.84 | 3.788 |
| NT-proBNP (pg/mL) | Total | 511 | 26.975 | 45.46 | 72.33 |
|  | Male | 321 | 22.02 | 39.56 | 67.98 |
|  | Female | 190 | 34.465 | 51.535 | 83.812 |

**Panel B. China-PAR risk and high-risk proportion within biomarker quartiles**

| Biomarker | Group | Quartile | n | Observed biomarker range | China-PAR risk, mean ± SD (%) | High-risk, n (%) [95% CI] | Trend in China-PAR risk, β (SE); P | Trend in high-risk proportion, χ² (df=1); P |
| --- | --- | --- | --- | --- | --- | --- | --- | --- |
| CysC | Total | Q1 | 128 | 0.63–0.96 | 7.63 ± 5.27 | 29 (22.7) [16.3–30.6] | 1.813 (0.226); <0.001 | 51.949; <0.001 |
|  |  | Q2 | 128 | 0.96–1.07 | 7.59 ± 4.21 | 30 (23.4) [16.9–31.5] |  |  |
|  |  | Q3 | 128 | 1.07–1.20 | 10.23 ± 6.41 | 52 (40.6) [32.5–49.3] |  |  |
|  |  | Q4 | 127 | 1.20–5.13 | 12.80 ± 6.49 | 80 (63.0) [54.3–70.9] |  |  |
|  | Male | Q1 | 81 | 0.78–1.00 | 9.44 ± 5.75 | 29 (35.8) [26.2–46.7] | 1.307 (0.308); <0.001 | 19.554; <0.001 |
|  |  | Q2 | 80 | 1.00–1.10 | 9.77 ± 6.99 | 29 (36.2) [26.6–47.2] |  |  |
|  |  | Q3 | 80 | 1.10–1.23 | 10.45 ± 5.40 | 32 (40.0) [30.0–51.0] |  |  |
|  |  | Q4 | 80 | 1.23–5.13 | 13.58 ± 6.37 | 57 (71.2) [60.5–80.0] |  |  |
|  | Female | Q1 | 48 | 0.63–0.92 | 5.81 ± 3.76 | 6 (12.5) [5.9–24.7] | 1.638 (0.293); <0.001 | 19.171; <0.001 |
|  |  | Q2 | 48 | 0.92–1.02 | 6.22 ± 2.96 | 6 (12.5) [5.9–24.7] |  |  |
|  |  | Q3 | 47 | 1.02–1.14 | 6.66 ± 4.11 | 8 (17.0) [8.9–30.1] |  |  |
|  |  | Q4 | 47 | 1.15–2.89 | 11.14 ± 6.15 | 24 (51.1) [37.2–64.7] |  |  |
| FPG | Total | Q1 | 128 | 3.73–5.03 | 7.66 ± 4.02 | 34 (26.6) [19.7–34.8] | 2.079 (0.222); <0.001 | 51.949; <0.001 |
|  |  | Q2 | 128 | 5.03–5.52 | 7.72 ± 4.76 | 31 (24.2) [17.6–32.3] |  |  |
|  |  | Q3 | 128 | 5.53–6.55 | 8.54 ± 4.93 | 35 (27.3) [20.4–35.6] |  |  |
|  |  | Q4 | 127 | 6.57–19.08 | 14.33 ± 7.32 | 91 (71.7) [63.3–78.8] |  |  |
|  | Male | Q1 | 81 | 3.73–5.03 | 8.88 ± 3.97 | 28 (34.6) [25.1–45.4] | 1.967 (0.297); <0.001 | 25.225; <0.001 |
|  |  | Q2 | 80 | 5.03–5.65 | 9.73 ± 5.62 | 31 (38.8) [28.8–49.7] |  |  |
|  |  | Q3 | 80 | 5.65–6.70 | 8.90 ± 4.25 | 25 (31.2) [22.2–42.1] |  |  |
|  |  | Q4 | 80 | 6.71–17.21 | 15.73 ± 8.01 | 63 (78.8) [68.6–86.3] |  |  |
|  | Female | Q1 | 48 | 4.29–5.02 | 5.54 ± 3.14 | 6 (12.5) [5.9–24.7] | 2.158 (0.274); <0.001 | 28.105; <0.001 |
|  |  | Q2 | 48 | 5.03–5.45 | 5.49 ± 2.83 | 4 (8.3) [3.3–19.6] |  |  |
|  |  | Q3 | 47 | 5.46–6.15 | 6.36 ± 2.60 | 6 (12.8) [6.0–25.2] |  |  |
|  |  | Q4 | 47 | 6.22–19.08 | 12.47 ± 6.11 | 28 (59.6) [45.3–72.4] |  |  |
| AC | Total | Q1 | 128 | 0.04–1.82 | 7.46 ± 4.38 | 28 (21.9) [15.6–29.8] | 1.604 (0.229); <0.001 | 31.950; <0.001 |
|  |  | Q2 | 128 | 1.84–2.75 | 9.09 ± 5.30 | 44 (34.4) [26.7–43.0] |  |  |
|  |  | Q3 | 128 | 2.78–3.80 | 8.77 ± 4.76 | 46 (35.9) [28.1–44.5] |  |  |
|  |  | Q4 | 127 | 3.81–24.73 | 12.92 ± 7.82 | 73 (57.5) [48.8–65.7] |  |  |
|  | Male | Q1 | 81 | 0.04–1.82 | 8.02 ± 3.96 | 20 (24.7) [16.6–35.1] | 1.977 (0.297); <0.001 | 26.241; <0.001 |
|  |  | Q2 | 80 | 1.84–2.71 | 10.55 ± 5.80 | 37 (46.2) [35.7–57.1] |  |  |
|  |  | Q3 | 80 | 2.73–3.86 | 9.83 ± 4.32 | 36 (45.0) [34.6–55.9] |  |  |
|  |  | Q4 | 80 | 3.86–24.73 | 14.86 ± 8.31 | 54 (67.5) [56.6–76.8] |  |  |
|  | Female | Q1 | 48 | 0.19–1.84 | 6.43 ± 4.89 | 8 (16.7) [8.7–29.6] | 0.999 (0.308); 0.001 | 7.216; 0.007 |
|  |  | Q2 | 48 | 1.86–2.86 | 6.58 ± 3.20 | 8 (16.7) [8.7–29.6] |  |  |
|  |  | Q3 | 47 | 2.89–3.79 | 7.26 ± 4.87 | 9 (19.1) [10.4–32.5] |  |  |
|  |  | Q4 | 47 | 3.79–10.27 | 9.55 ± 5.66 | 19 (40.4) [27.6–54.7] |  |  |
| NT-proBNP | Total | Q1 | 128 | 5.02–26.97 | 8.75 ± 5.67 | 35 (27.3) [20.4–35.6] | 0.959 (0.236); <0.001 | 21.811; <0.001 |
|  |  | Q2 | 128 | 26.98–45.46 | 8.37 ± 4.59 | 36 (28.1) [21.1–36.5] |  |  |
|  |  | Q3 | 128 | 45.65–72.36 | 9.57 ± 5.98 | 53 (41.4) [33.2–50.1] |  |  |
|  |  | Q4 | 127 | 72.75–1976.72 | 11.55 ± 7.25 | 67 (52.8) [44.1–61.2] |  |  |
|  | Male | Q1 | 81 | 5.45–22.02 | 8.91 ± 5.48 | 23 (28.4) [19.7–39.0] | 1.471 (0.306); <0.001 | 22.300; <0.001 |
|  |  | Q2 | 80 | 22.81–39.56 | 10.04 ± 4.60 | 33 (41.2) [31.1–52.2] |  |  |
|  |  | Q3 | 80 | 39.71–67.98 | 10.71 ± 6.06 | 39 (48.8) [38.1–59.5] |  |  |
|  |  | Q4 | 80 | 68.12–1976.72 | 13.59 ± 7.90 | 52 (65.0) [54.1–74.5] |  |  |
|  | Female | Q1 | 48 | 5.02–34.24 | 6.97 ± 5.31 | 9 (18.8) [10.2–31.9] | 0.757 (0.311); 0.016 | 8.067; 0.005 |
|  |  | Q2 | 48 | 35.14–51.54 | 6.08 ± 3.72 | 4 (8.3) [3.3–19.6] |  |  |
|  |  | Q3 | 47 | 51.56–83.95 | 7.85 ± 5.27 | 13 (27.7) [16.9–41.8] |  |  |
|  |  | Q4 | 47 | 83.96–833.18 | 8.91 ± 4.67 | 18 (38.3) [25.8–52.6] |  |  |

**Note:** Quartiles were defined separately within the total cohort and within each sex subgroup, consistent with the analysis used for Figure 4. P25, P50, and P75 denote the 25th, 50th, and 75th percentiles, respectively. High risk was defined as a China-PAR 10-year ASCVD risk ≥10%. The trend in continuous China-PAR risk was tested by linear regression using quartile number (1–4) as an ordinal term; β is the change in risk percentage points per quartile. The trend in high-risk proportion was assessed using a Cochran–Armitage trend test. AC, atherogenic coefficient; CysC, cystatin C; FPG, fasting plasma glucose; NT-proBNP, N-terminal pro-B-type natriuretic peptide; CI, confidence interval.
